# Management of refractory Mycobacterium abscessus sternal infection following reoperative cardiac surgery

**DOI:** 10.1002/ccr3.4027

**Published:** 2021-03-09

**Authors:** Brandon M. Wojcik, John D. Mitchell, Tae Chong, Jessica Y. Rove

**Affiliations:** ^1^ Division of Cardiothoracic Surgery Department of Surgery University of Colorado Aurora CO USA; ^2^ Division of Plastic Surgery Department of Surgery University of Colorado Aurora CO USA

**Keywords:** cardiothoracic surgery, infectious diseases, mycobacterium abscessus, nontuberculous mycobacteria, postoperative infection

## Abstract

*Mycobacterium abscessus* surgical site infections are rare, but notoriously difficult to treat. Eradication requires aggressive surgical resection, removal of foreign material, prolonged antibiotics, and consideration of delayed reconstruction.

## Graphical abstract

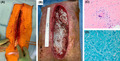

## 1 INTRODUCTION

*Mycobacterium abscessus* is a rare cause of surgical site infections, but is notoriously drug‐resistant. Providers must understand the guideline‐directed therapy required to prevent recurrence. We present a case of recurrent postoperative *Mycobacterium abscessus* sternal infection that was eradicated with aggressive surgical resection, topical/systemic antibiotic therapy, and delayed sternal reconstruction.

Nontuberculous mycobacteria (NTM) are a heterogenous group of over 150 species found ubiquitously in the environment.^1^ They are opportunistic pathogens, with a subset causing disease in humans with impaired host defenses and in areas of pre‐existing tissue injury. NTM are emerging causes of healthcare‐associated infections following contact with contaminated water, aerosols, or medical equipment. During the last decade, contamination of heater‐cooler units used in cardiac bypass surgery was implicated in a global outbreak of over 100 postoperative infections from *Mycobacterium chimaera*.^1^ *Mycobacterium abscessus* is a rapid‐growing species that is notoriously drug‐resistant and requires aggressive surgical and medical management.^2^ This report describes a novel management strategy to treat a patient with a refractory postoperative *Mycobacterium abscessus* sternal infection.

## 2 CASE REPORT

A 67‐year‐old man with a history of coronary artery bypass grafting underwent a mitral and tricuspid valve repair through a redo sternotomy performed at an outside hospital. He was readmitted one month following surgery with serous drainage from his wound. Imaging demonstrated inferior sternal widening with fractured wires and a substernal fluid collection. Operative exploration revealed no purulent fluid, cultures were obtained, and the fluid collection was felt to be a result of the dehiscence. The sternum was closed with wires, reinforced with lower sternal plates under myocutaneous flaps, and the skin was left open with a wound vac. While undergoing vac changes, his sternal and soft tissue cultures grew *Mycobacterium abscessus* that was macrolide sensitive. Infectious disease was consulted, and he was placed on an appropriate triple antibiotic regimen.^3^ The patient returned to the operating room with the assistance of plastic surgery for removal of the lower sternal hardware, sternal debridement, bilateral pectoralis advancement flaps, and skin closure. Cultures taken during this final operation eventually grew *Mycobacterium abscessus*. After extensive multidisciplinary discussions, further chest wall resection was felt to be high‐risk. A referral was placed to a center with expertise and he was discharged on oral azithromycin, intravenous amikacin, and intravenous imipenem.^3^

During the ensuing months, he was monitored closely as an outpatient. Drainage from the inferior wound recurred four months following discharge, prompting referral to our institution. Imaging revealed a substernal fluid collection with a draining sinus tract, three upper sternal wires, and sternal sclerosis (Figure postoperative1). Transthoracic echocardiogram demonstrated competent valve repairs and no vegetations. Coronary angiography demonstrated a patent left internal mammary artery (LIMA) graft crossing directly beneath the left upper sternum. The case was reviewed with colleagues in infectious disease and plastic surgery. The decision was made to pursue radical surgical resection, removal of all hardware, serial debridements, and delayed sternal reconstruction after operative cultures cleared. Preoperative nutritional assessment demonstrated adequate nourishment. His antibiotic regimen was expanded to four agents including oral azithromycin, intravenous imipenem, intravenous tigecycline, and intravenous clofazimine.

**FIGURE 1:**
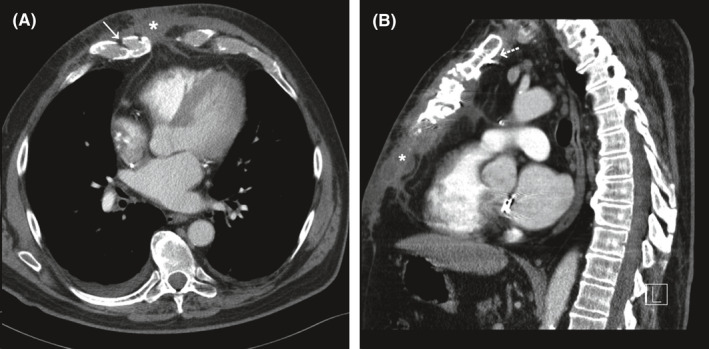
Chest computed tomography axial (A) and sagittal (B) sections demonstrating recurrent infection. White arrow indicates sternal sclerosis. White asterisk indicates substernal fluid collection tracking to the inferior wound. White dashed arrow demonstrates a retained sternal wire

**FIGURE 2:**
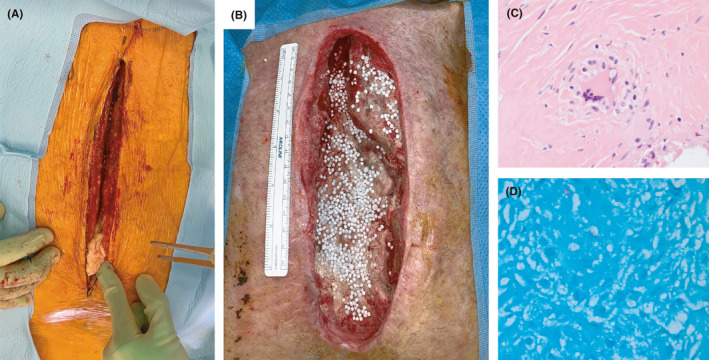
Intraoperative images demonstrating the (A) inferior sternal abscess and (B) near complete sternectomy with placement of amikacin antimicrobial beads. Microscopic histopathology findings demonstrating (C) granuloma formation in the fibrotic tissue surrounding inferior sternum and (D) acid‐fast stain positive for microorganisms within the granuloma

He was taken to the operating room seven months following his initial valve repair. The sternal scar was excised. An abscess was encountered in the soft tissue at the lower third of the sternal incision that tracked through the pectoralis muscle flaps, inferior sternum, and into the mediastinum (Figure 2A). The inferior sternum was sclerotic and grossly diseased. Tissue cultures were obtained, the remaining sternal wires were removed, and a redo sternotomy with nearly complete sternectomy was performed. The left manubrium and upper sternum were not removed as they were remote from the nidus of infection, appeared normal, and we did not want to risk injury to the patent LIMA graft adherent to its underside (Figure 2B). The abscess cavity was debrided to bleeding tissue except for that directly over the heart. A pulse lavage system (Stryker Inc, Mahwah, NJ) was used to irrigate all exposed mediastinal surfaces with bacitracin (50,000 U/3 L 0.9% normal saline). Absorbable synthetic calcium sulfate beads (Stimulan, Biocomposites, UK) infused with amikacin (5 g in 50 cc of beads) were placed in the wound bed (Figure 2B). A temporary wound vac was placed, and the patient was admitted to the cardiac surgery ward.

**FIGURE 3:**
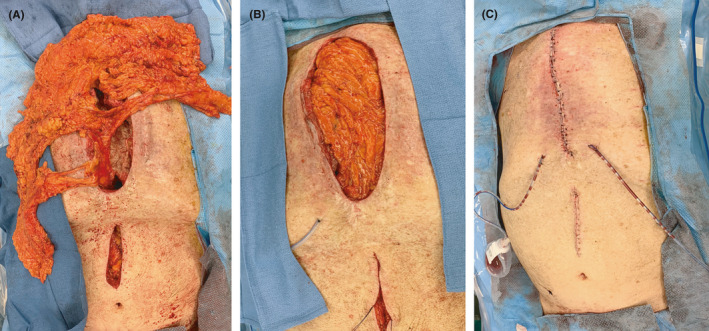
Intraoperative images from delayed sternal reconstruction demonstrating the (A) omental flap tunneled into the mediastinum and (B) positioned in the surgical bed prior to (C) closure of bilateral skin advancement flaps

Tissue staining revealed granulomas with acid‐fast bacilli in the connective tissue surrounding the lower sternum, but no active infection of the bone (Figure 2C‐D). Weekly operative debridements were performed while obtaining new cultures and replacing the amikacin beads. At five weeks following initial sternectomy, cultures showed no growth. Wound closure required an omental flap to fill the spatial defect. A pedicled omental flap supplied by the right gastroepiploic artery was tunneled through the diaphragm into the mediastinum (Figure 3A‐B). Plastic surgery elevated bilateral full thickness skin flaps to the level of the remaining pectoralis major fascia. Indocyanine green fluorescence angiography confirmed vascularization of both omental and skin flaps. The skin flaps were advanced over the omentum and closed primarily over drains (Figure 3C).

The remainder of the patient's hospital course was uncomplicated, and he was discharged home after removal of both drains. He was monitored closely as an outpatient on the aforementioned guideline‐directed antibiotic regimen.^3^ This was continued for four months following sternectomy, the recommended course for serious complex soft tissue infections.^3^ At one year following surgery, he has no evidence of recurrent infection.

## 3 COMMENT

Postcardiotomy NTM infections are rare, but when diagnosed, the infections are associated with high morbidity and mortality.^1^ Diagnosis is often delayed given their slow growth in cultures and a low index of suspicion. This case emphasizes several important considerations in the management of this complex condition.

First, successful management of sternal and mediastinal NTM infection requires a multidisciplinary approach including infectious disease expertise for optimal medical therapy and surgeons for both resection and reconstruction. There should be a low threshold for referral to a center with experience in the management of complex NTM infections.

Second, guideline‐directed antimicrobial therapy for NTM infections requires multidrug regimens and a combination of oral and intravenous agents to obtain appropriate tissue concentrations.^3^ In an effort to deliver a higher concentration of antimicrobials to the affected site, we utilized amikacin beads in addition to systemic therapy. While amikacin beads have previously been described in the treatment of NTM infections, this is, to the best of our knowledge, the first reported use in a NTM sternal infection.^4^

Finally, optimal treatment requires aggressive surgical debridement and removal of foreign material. The surgical bed should be monitored with cultures and reconstruction delayed until after the infection has cleared. Large defects are best filled using autologous tissue flaps. The omentum was an excellent choice for our patient given the size of the defect and failure of prior pectoralis muscle flaps.

In conclusion, we report a rare case of refractory *Mycobacterium abscessus* sternal infection following reoperative cardiac surgery. A treatment plan was devised by a multidisciplinary team and the infection was eradicated with aggressive surgical resection, a combination of local and systemic antimicrobial therapy, and delayed sternal reconstruction.

## CONFLICT OF INTEREST

None declared.

## AUTHOR CONTRIBUTIONS

BW, JM, TC, and JR: were actively involved in the clinical care of the patient. BW and JR: wrote the manuscript. JM and TC revised the manuscript.

## ETHICAL APPROVAL

Case reports of 3 or less patients are not subject to IRB regulations at our institution and were found by our COMIRB to exempt after submission.

## PATIENT CONSENT

Written consent was obtained by the patient and a formal waiver was signed.

## Data Availability

No data were generated or analyzed in the presented research.
